# A Novel Video Compression Approach Based on Two-Stage Learning

**DOI:** 10.3390/e26121110

**Published:** 2024-12-19

**Authors:** Dan Shao, Ning Wang, Pu Chen, Yu Liu, Lin Lin

**Affiliations:** 1 School of Computer Science and Technology, Changchun University, Changchun 130022, China; shaodan@ccu.edu.cn (D.S.); 220701280@mails.ccu.edu.cn (N.W.); 230702296@mails.ccu.edu.cn (P.C.); 220701271@mails.ccu.edu.cn (Y.L.); 2 School of Software Technology, Dalian University of Technology, Dalian 116024, China

**Keywords:** image compression, video compression, motion estimation, optical flow estimation

## Abstract

In recent years, the rapid growth of video data posed challenges for storage and transmission. Video compression techniques provided a viable solution to this problem. In this study, we proposed a bidirectional coding video compression model named DeepBiVC, which was based on two-stage learning. Firstly, we conducted preprocessing on the video data by segmenting the video flow into groups of continuous image frames, with each group comprising five frames. Then, in the first stage, we developed an image compression module based on an invertible neural network (INN) model to compress the first and last frames of each group. In the second stage, we designed a video compression module that compressed the intermediate frames using bidirectional optical flow estimation. Experimental results indicated that DeepBiVC outperformed other state-of-the-art video compression methods regarding PSNR and MS-SSIM metrics. Specifically, on the VUG dataset at bpp = 0.3, DeepBiVC achieved a PSNR of 37.16 and an MS-SSIM of 0.98.

## 1. Introduction

Imaging technologies have made great strides in improving the quality of video capture over the decades. However, the rapid growth of video data has created new challenges in storage and transmission [1]. The emergence of video compression techniques provided a solution to this problem. Video compression methods are mainly categorized into intra-frame compression, inter-frame compression, and hybrid coding compression [2]. Intra-frame compression usually employs image compression techniques such as Huffman coding [3], where each video frame is processed individually to eliminate statistical redundancy in the image data [4]. In contrast, inter-frame compression reduces spatial redundancy between consecutive frames by predicting motion information between the preceding and following frames [5]. Optical flow estimation is a commonly used technique for motion compensation. Hybrid coding compression methods eliminate redundant data by dividing the image into pixel macroblocks and matching similar macroblocks intra- and inter-frame [6]. The need for video compression methods arises from the challenge of balancing compression efficiency, computational complexity, and maintaining high-quality video output.

With the rapid advancement of deep learning technology, its application areas are broadening, particularly in the field of video compression [7,8,9]. In 2016, Ballé et al. introduced a pioneering image compression model based on convolutional neural networks (CNNs), utilizing an encoder-decoder framework specifically designed for image compression [10]. Following this, Toderici et al. proposed a recurrent neural network (RNN)-based approach to image compression that emphasized capturing pixel dependencies [11]. More recently, image compression utilizing generative adversarial networks (GANs) has gained significant attention. Rippel and Bourdev introduced an integrated and optimized GAN-based method [12], which balanced the quality of reconstructed images with the bit rate through adversarial training.

In the realm of video compression, deep learning is progressively being employed to tackle the challenges associated with optical flow estimation. Optical flow estimation fundamentally revolves around pixel-level matching, with the goal of identifying corresponding pixel points across adjacent frames. Flownet was the first model to leverage CNNs for optical flow estimation [13]. Subsequently, SpyNet employed CNNs to compute optical flows using a spatial pyramid structure [14]. Based on SpyNet, Lu et al. introduced a deep video compression (DVC) framework [15]. Additionally, Yang et al. proposed a recurrent learned video compression (RLVC) model, which also incorporated SpyNet for optical flow estimation and utilized a recursive auto-encoder (RAE) along with a recursive probabilistic model (RPM) to achieve low-latency video compression with sequential motion compensation [16]. However, it is worth noting that all these models depended on the pre-trained SpyNet optical flow estimation network, which posed a limitation: since SpyNet was developed independently, it cannot be co-trained with a video compression model.

To tackle the issues mentioned above, we presented a bidirectional coding video compression model named DeepBiVC, which was based on two-stage learning. Firstly, we preprocessed the video data by segmenting the video into groups of continuous image frames. Then, in the first stage, we developed an image compression module based on an invertible neural network (INN) to compress each group’s first and last frames. In the second stage, we designed a video compression module that compressed the intermediate frames using bidirectional optical flow estimation. Experimental results indicated that DeepBiVC outperformed other state-of-the-art video compression models.

Our key contributions are as follows:

(1) We proposed a two-stage learning strategy for video compression. In the first stage, the image compression network was trained independently for the first and last frames. While in the second stage, the video compression network was trained for the intermediate frames.

(2) We incorporated the INN structure into the video compression model for the first time. Its invertibility allowed the same network to be used for both image compression and reconstruction, thus reducing the complexity of the model.

(3) We introduced an optical flow estimation network that utilizes adjacent image features to achieve global matching for motion estimation, enabling joint optimization with video compression.

The rest of the paper was organized as follows. Section 2 reviewed related work on image and video compression. Section 3 presented the video compression method proposed in this study. Section 4 demonstrated the experimental results of the proposed method. Section 5 discussed the advantages and limitations of the method. Finally, Section 6 provides the conclusion of the research.

## 2. Related Work

### 2.1. Image Compression

The International Standards Organization (ISO) developed the first international image compression standard, JPEG [17], in 1992. This standard was primarily used for compressing continuous-tone still images, including grayscale and color images. The discrete cosine transform (DCT) [18] converted pixel values from the spatial domain into coefficients in the frequency domain. It then compressed the quantized DCT coefficients using Huffman coding [3], thereby eliminating redundancy in the image and color data. With its high compression ratio and excellent performance, the JPEG standard was widely adopted.

Google introduced the WebP format [19]. WebP employed arithmetic coding for entropy coding and achieved higher compression ratios than JPEG, although its encoding time was eight times longer. Subsequently, in 2015, the BPG format [20] was proposed to deliver higher quality images than JPEG at the same file size. BPG was based on a subset of the H.265/HEVC standard, utilizing intra-frame prediction for pixel block matching to eliminate spatial redundancy and compressing data with context-adaptive binary arithmetic coding (CABAC) [21].

With the continuous advancement of deep learning, its applications in image compression emerged. Ballé et al. proposed an end-to-end CNN-based image compression framework [10]. This framework employed CNNs and generalized division normalization (GDN) [22] to create a nonlinear transform in place of the traditional DCT. Experimental results demonstrated that this method outperformed JPEG in terms of compression performance. The main role of GDN was to normalize the activation values of each neuron, enabling comparisons with the activation values of other neurons in the same layer. This normalization enhanced the suitability of these values for processing in the subsequent layers of the neural network.

(1) $$Y_{i}=\frac{X_{i}}{{\left(b_{i}+\sum_{j}c_{ij}X_{j}^{2}\right)}^{a}}$$

where $Y_{i}$ was the normalized output, and $X_{i}$ was the *i*-th component of the input signal $X_{j}$, where i<j. $b_{i}$ was an offset parameter that can be learned to ensure the stability of the normalization process and to prevent division by zero errors, $c_{ij}$ was a weight parameter that can be learned to indicate the relationship between the input signal $X_{j}$ and the output $Y_{i}$, and *a* was an adjustable hyper-parameter that was usually set to 0.5.

In recent years, GANs have led to the development of image compression performance. In 2015, Denton et al. combined GAN and conditional GAN (cGAN) [23] to propose Lap-GAN [24], which applied a pyramid structure to enable the model to reconstruct higher-resolution images. Subsequently, in 2017, Toderici et al. introduced a full-resolution image compression method [11] by applying RNNs for the first time in the field of image compression. More recently, Xiao et al. applied INNs [25,26] to image compression and proposed an invertible scaling network (IRN) [27]. This network created an accurate mapping between high-resolution and low-resolution images. The IRN not only preserved more image details during compression but also accurately restored the original image during decompression.

### 2.2. Video Compression

Early efforts in video compression focused on encoding pixel data using Huffman coding and arithmetic coding techniques, known as the entropy coding stage. To further improve compression efficiency, classical transform coding techniques such as the discrete fourier transform (DFT) [28], Hadamard transform [29], and DCT were introduced and widely adopted. These transform coding techniques significantly enhanced the compression ratio by converting the video signal from the time or spatial domain into the frequency or transform domain, which was more suitable for compression while maintaining signal quality as much as possible.

**Figure 1:**
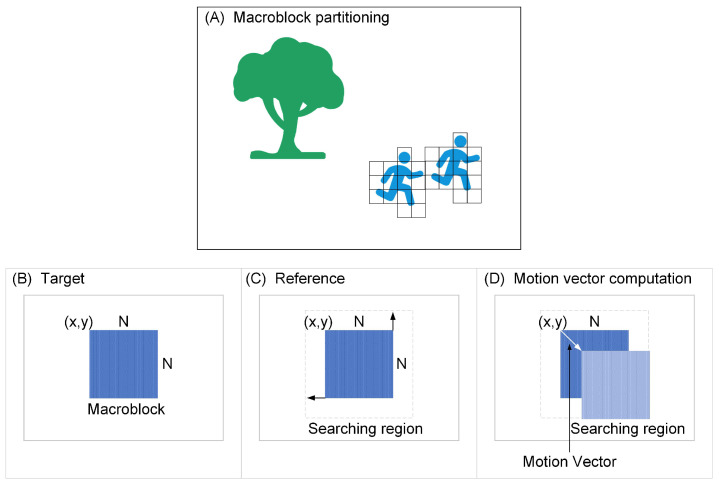
Block-based motion estimation method. (**A**) The image is divided into macroblocks. (**B**) A macroblock in the target frame. (**C**) Reference frame, where the macroblock searched for similar blocks. (**D**) The macroblocks in the target frame are found to be the most similar blocks within the specified search range by using a block matching algorithm in the reference frame.

In 1988, the ITU-T organization released the pioneering H.261 standard [30]. H.261 calculated the similarity of consecutive frames using inter-frame prediction and combined it with motion vectors for motion compensation, effectively eliminating spatial redundancy. The ITU-T then introduced the H.263 standard [31] in 1996, which employed a hybrid coding framework based on block-based inter-frame prediction combined with the DCT. As shown in Figure 1, the block-based motion estimation method divided the image into macroblocks and used a block-matching algorithm to find similar macroblocks in neighboring frames. The motion vector was determined by the amount of motion variation between the original block and the matched block. Building on these advancements, the ITU-T video coding expert group and the ISO/IEC motion picture expert group jointly proposed H.264/AVC [32] in 2003. H.264/AVC divided the image into fixed 16×16 pixel blocks for intra-frame and inter-frame prediction and employed entropy coding techniques that were faster than conventional DCTs, such as CABAC and context adaptive variable length coding (CAVLC). In 2013, the H.265/HEVC standard [33] was released. This standard continued to use the classic block-based hybrid video coding framework but offered more flexible macroblock division compared to H.264/AVC, allowing divisions into macroblocks of varying sizes (from 8×8 pixels to 64×64 pixels). This flexibility enabled more accurate matching of image content. Additionally, H.265/HEVC enhanced CABAC from H.264/AVC to increase both encoding efficiency and decoding speed.

**Figure 2:**
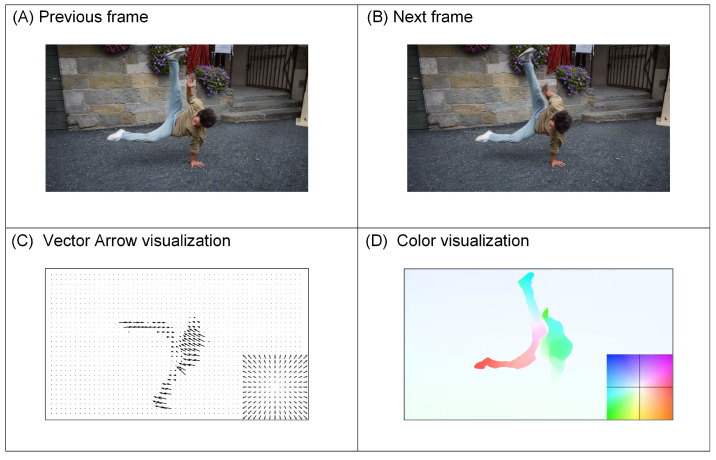
Optical flow estimation method. (**A**) The previous frame in the adjacent frame. (**B**) The next frame in the adjacent frame. (**C**) Vector arrow visualization of optical flow. The direction and length of the arrow represent the direction and magnitude of the optical flow vector, respectively. (**D**) Color visualization of optical flow, where hue indicates the direction of motion, while saturation indicates the speed or magnitude of movement.

In recent years, several computational video compression methods have been proposed. Lu et al. introduced the first end-to-end video compression framework [15]. This framework utilized optical flow for motion estimation and employed two separate self-encoders to compress motion and residual information. The optical flow estimation captured the transformation relationship between two adjacent frames, as illustrated in Figure 2. The optical flow between these frames can be intuitively represented through vector arrow visualization and color visualization.

The recently proposed recurrent learning video compression method, RLVC [16], employed a recursive structure to compress successive frames. It featured an RAE that utilized information from multiple preceding frames to compress the motion and residuals of the current frame, alongside an RPM for entropy coding. Another approach was the feature space video coding network (FVC) [34], which transformed the image into feature space using a feature extraction module composed of convolutional layers and residual blocks. Key operations for video compression—such as motion estimation, motion compression, motion compensation, and residual compression—were conducted in this feature space. Additionally, Djelouah et al. introduced the hierarchical learning video compression model, hierarchical learned video compression (HLVC) [35]. This model constructed three hierarchical quality layers and a recurrent enhancement network, segmenting the video frame sequence into three quality layers and applying different quality compression methods for each layer. This approach minimized unnecessary computational complexity while enhancing compression efficiency.

## 3. Proposed Method

### 3.1. Dataset

The data used in this study was sourced from two datasets. The first dataset, referred to as Dataset A, was obtained from Vimeo-90K (http://toflow.csail.mit.edu, accessed on 18 June 2023) [36]. This dataset contains 89,800 video clips, with each clip consisting of 7 frames at a resolution of 448 × 256. Before training, we randomly cropped the video sequences to a resolution of 256 × 256. Additionally, we collected a second dataset for external validation, referred to as Dataset B, from UVG1080p (https://ultravideo.fi/dataset.html, accessed on 20 June 2023) [37]. This dataset includes 7 videos, all at a resolution of 1920 × 1080. In our experiments, Dataset A was split into a training dataset and a testing dataset in an 80:20 ratio.

### 3.2. The Overall Architecture of the Model

**Figure 3:**
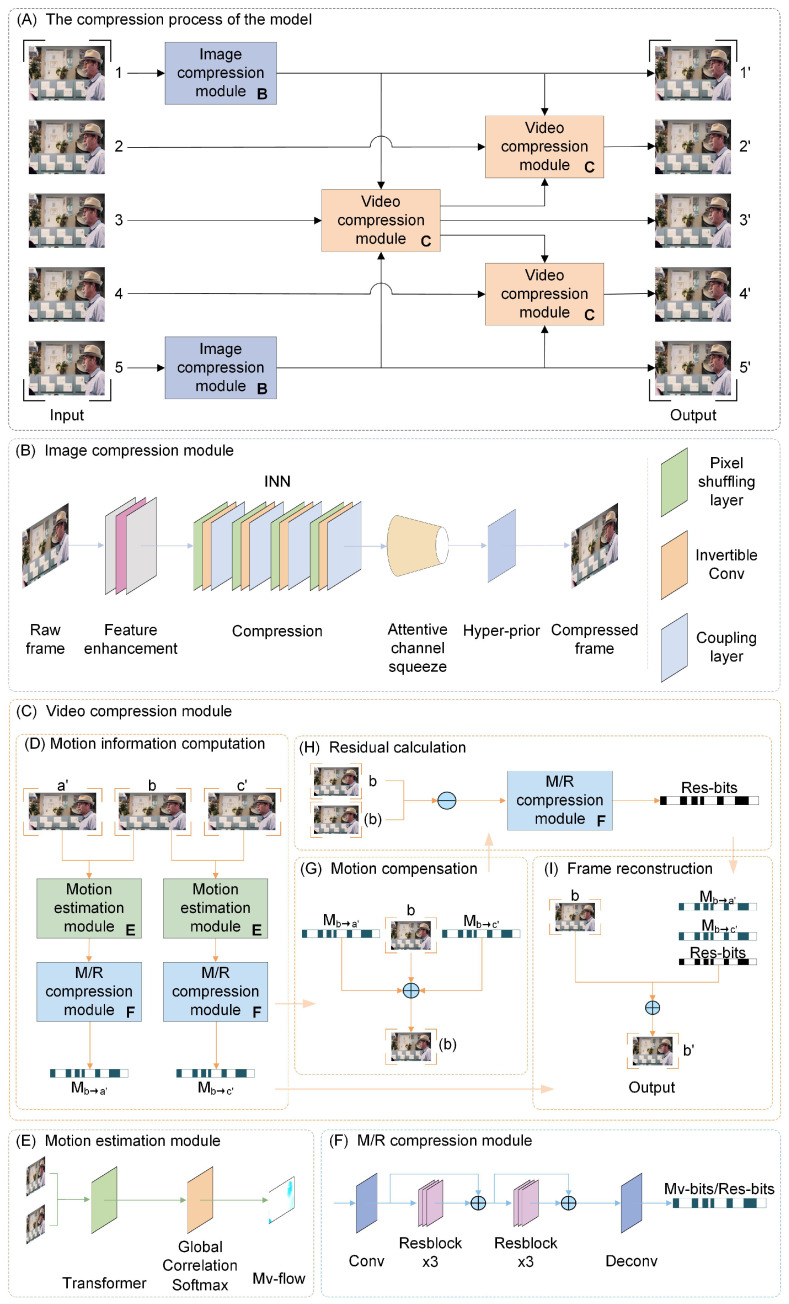
Overall structure of the model. (**A**) The overall compression process of DeepBiVC, where the input was the original frame sequence, and the output was the compressed frame sequence. (**B**) The image compression process. (**C**) The video compression process. (**D**) Motion information computation, where *b* represented the original frame in the input, and $a^{\prime }$ and $c^{\prime }$ were the compressed adjacent frames. (**E**) The calculation of motion vectors between adjacent frames was performed using a motion estimation network; the input consisted of two adjacent frame images, with Mv-flow denoting the motion vectors. (**F**) The motion estimation results in Mv-flow and the residual between the predicted frame (b) and the original frame *b* were encoded and compressed, resulting in the compressed motion vector Mv-bits and the residual Res-bits. (**G**) The current frame *b* was predicted using the bidirectional motion vectors $M_{a^{\prime }\to b}$ and $M_{c^{\prime }\to b}$. (**H**) The residual between the predicted frame (b) and the original frame *b* was calculated, followed by the compression of this residual. (**I**) The original frame was reconstructed using the residual Res-bits and the bidirectional motion vectors $M_{a^{\prime }\to b}$ and $M_{c^{\prime }\to b}$, resulting in the compressed reconstructed frame $b^{\prime }$. M/R motion and residual.

The proposed DeepBiVC performed video compression using a two-stage learning strategy: the first stage focused on image compression for the first and last frames, while the second stage handled the video compression for the intermediate frames. The structure is illustrated in Figure 3.

Initially, we preprocessed the video data by segmenting it into continuous image frames at a rate of 60 frames per second. Subsequently, we organized the sequence of image frames into groups, with each group comprising a total of five frames, labeled as 1, 2, 3, 4, and 5.

For the first frame 1 and the last frame 5, we proposed an image compression module based on INN, which produced the compressed outputs 1′ and 5′. In contrast, for the intermediate frames, we developed a video compression module based on optical flow estimation. Firstly, the compressed frames 1′ and 5′, along with the original frame 3, were input into this module to extract bidirectional motion information from 1′ to frame 3 and 5′ to frame 3. This information then was compressed by the motion and residual (M/R) compression module. Motion compensation was utilized to generate the predicted frame (3). The residual between the predicted frame (3) and the original frame 3 was calculated and subsequently compressed to reconstruct and produce the compressed frame 3′. Next, the frames 1′, 3′, and the original frame 2 were fed into the video compression module for processing, similar to the above process, resulting in the compressed second frame 2′. Finally, the frames 3′, 5′, and the frame 4 were input into the video compression module to produce the compressed fourth frame 4′. Through this process, a set of compressed frames 1′, 2′, 3′, 4′, and 5′ was obtained.

### 3.3. Image Compression Module

The module functioned to compress the first and last frames of the groups. It comprised four parts: feature enhancement, compression, attention channel squeeze, and hyper-prior, as shown in Figure 3B. Due to its invertibility, where the positive encoding represented the compression process, and the reverse decoding was used for decompression.

#### 3.3.1. Feature Enhancement

**Figure 4:**
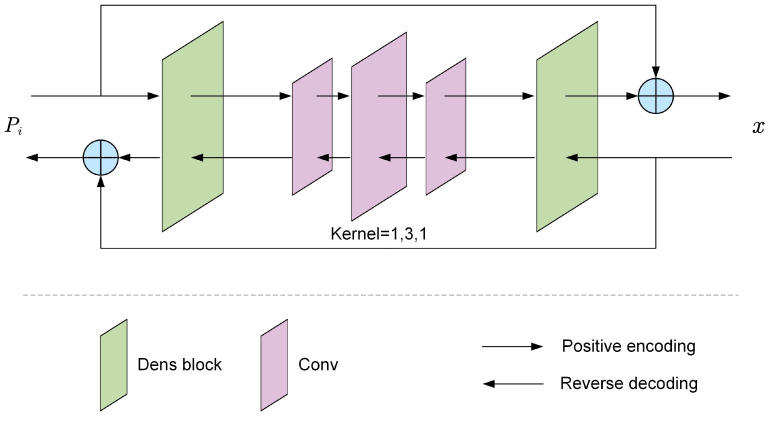
Feature enhancement module. It consisted of two dense blocks and three convolutional layers with different kernel sizes. The positive encoding process extracted features *x* from the image $P_{i}$, while the reverse decoding process reconstructed the original image $P_{i}$ from the features *x*.

To enhance the nonlinear representation capability of the network, we employed a feature enhancement module that included two dense blocks [38] and three convolutional layers with kernel sizes of 1, 3, and 1. The structure was illustrated in Figure 4. During the positive encoding process, the input was an image frame $P_{i}\in R^{3\times H\times W}$, while the output became $x\in R^{3\times H\times W}$, where *H* and *W* denoted the pixels in the vertical and horizontal direction, respectively. The three convolutional layers were utilized to improve the feature representation capability of the input images, while the dense blocks were employed to address the nonlinear mapping between the images and their features. In the dense block, the output $x_{l}$ for layer *l* can be expressed as:

(2) $$x_{l}=LeakyReLu\left(\sum_{m=0}^{M}\sum_{n=0}^{N}concat\left[x_{0},x_{1},\ldots ,x_{\left(l-1\right)}\right]\cdot W_{l}\left(m,n\right)\right)$$

(3) $$LeakyReLu\left(x\right)=\left\{\begin{array}{cc} x, & x>0\\ \alpha x, & x⩽0 \end{array}\right.$$

where $concat[x_{0},x_{1},\ldots ,x_{\left(l-1\right)}]$ represented the concatenation of the outputs from the previous l−1 layers. LeakyReLu(·) was the activation function, where α=0.01 was used to adjust the zero gradient problem for negative values of the input *x* with a tiny slope. $W_{l}\left(m,n\right)$ denoted the convolution kernel of the 2D convolution function at layer *l*, where (m,n) indicated the position of the kernel.

#### 3.3.2. Compression

This paper employed an INN to compress and decompress the first and last frames. The compression process transformed the original image from the spatial domain into the frequency domain, while the decompression process reconstructed the frequency features back into the original image’s spatial representation. Each INN structure consisted of four invertible blocks, with each block containing one down-scaling layer and three coupling layers with kernel size of *k*. The down-scaling layer comprised a pixel shuffling layer [39] and an invertible 1×1 convolution. Each down-scaling layer reduced the resolution of the input tensor by a factor of 2 and the channel dimension by a factor of 4. After traversing four invertible blocks, the input resolution was downscaled to 1/24 of its original size. In particular, the input tensor was $x\in R^{3\times H\times W}$. After passing through the downscaling layer, the output *d* was produced, where $d\in R^{\left(3\times 4^{4}\right)\times \frac{H}{2^{4}}\times \frac{W}{2^{4}}}$:

(4) $$d\left(rh+i,rw+j,c\right)=x\left(h,w,c\cdot r^{2}+i\cdot r+j\right)$$

where (h,w) were the position indices of the input tensor *x*, *c* was the output channel index, and *i*, *j* were the indices within the range of the scaling factor *r*.

The kernel size of the coupling layer of the four invertible blocks was set to k=5,5,3,3. The input to the affine coupling layer was $d=u_{1:A}^{i}$. The variable *a* represented a random value between 0 and *A*. At *a*, the input was divided into two segments, yielding the output $I=u_{1:A}^{\left(i+1\right)}$, where *I* denoted the output of the affine coupling layer:

(5) $$u_{1:a}^{\left(i+1\right)}=u_{1:a}^{\left(i\right)}⊙exp\left[\sigma_{a}\left(g_{2}\left(u_{a+1:A}^{\left(i\right)}\right)\right)\right]+h_{2}\left(u_{a+1:A}^{\left(i\right)}\right)$$

(6) $$u_{a+1:A}^{\left(i+1\right)}=u_{a+1:A}^{\left(i\right)}⊙exp\left[\sigma_{a}\left(g_{1}\left(u_{1:a}^{\left(i+1\right)}\right)\right)\right]+h_{1}\left(u_{1:a}^{\left(i+1\right)}\right)$$

where *i* denoted the current affine coupling layer, ⊙ represented the Hadamard product, exp[·] was the exponential function, and $\sigma_{a}\left(\cdot \right)$ was the centered sigmoid function. The Functions *g* and *h* were referred to as feedforward functions.

Conversely, when the coupling layer received the input $u_{1:A}^{\left(i+1\right)}$ at the same split position *a*, the affine coupling layer can provide a perfect inverse $u_{1:A}^{i}$:

(7) $$u_{a+1:A}^{i}=\left(u_{a+1:A}^{\left(i+1\right)}-h_{1}\left(u_{1:a}^{\left(i+1\right)}\right)\right)⊙exp\left[-\sigma_{a}\left(g_{1}\left(u_{1:a}^{\left(i+1\right)}\right)\right)\right]$$

(8) $$u_{1:a}^{i}=\left(u_{1:a}^{\left(i+1\right)}-h_{2}\left(u_{a+1:A}^{\left(i\right)}\right)\right)⊙exp\left[-\sigma_{a}\left(g_{2}\left(u_{a+1:A}^{\left(i\right)}\right)\right)\right]$$

#### 3.3.3. Attentive Channel Squeeze

A channel squeezing layer was introduced to decrease the total number of pixels in the input tensor. This layer integrated an attention mechanism to automatically adjust the importance of each channel. Given a compression ratio α and the input tensor $I\in R^{C\times H\times W}$, the channel squeezing layer reshaped the input *I* into $C\in R^{\left(C\times \alpha^{2}\right)\times \frac{H}{\alpha }\times \frac{W}{\alpha }}$, which was then fed into the attention module. The attention module utilized a bidirectional attention mechanism, consisting of two attention blocks that managed forward and backward propagation.

(9) $$y=forw_{attention}\left(C\right)=softmax\left(\frac{W_{Q}\cdot C\cdot {\left(W_{K}\cdot C\right)}^{T}}{\sqrt{d_{k}}}\right)\cdot \left(W_{V}\cdot C\right)$$

(10) $$C=back_{attention}\left(y\right)=softmax\left(\frac{W_{Q}^{\prime }\cdot y\cdot {\left(W_{K}^{\prime }\cdot y\right)}^{T}}{\sqrt{d_{k}}}\right)\cdot \left(W_{V}^{\prime }\cdot y\right)$$

where $W_{Q}$, $W_{K}$, $W_{V}$ were the parameter matrices in the forward attention mechanism, and $W_{Q}^{\prime }$, $W_{K}^{\prime }$, $W_{V}^{\prime }$ were the parameter matrices in the backward attention mechanism. $\sqrt{d_{k}}$ was a scaling factor that prevented the dot product from becoming excessively large.

#### 3.3.4. Hyper-Prior

The application of the hyper-prior module helped eliminate redundant information in the latent representation, facilitating more accurate entropy coding and further enhancing compression efficiency. In this paper, we adopted the hyper-prior proposed by Minnen et al. [40], which employed a Gaussian distribution with mean and scale. First, the latent features *y* output by the channel squeezing layer were parameterized to obtain *z*, and then *z* was quantized to produce $z^{\prime }$:

(11) $$z^{\prime }=h_{s}\left(z\right)=h_{s}\left(h_{a}\left(y\right)\right)$$

where $h_{a}$ was the analytical Gaussian transformation applied to *y*, $h_{s}$ stood for the synthetic Gaussian transformation applied to *z*.

(12) $$p_{y^{\prime }|z^{\prime }}\leftarrow \left\{h_{s}\left(z^{\prime }\right),C_{m}\left(y^{\prime }\right)\right\}$$

where $h_{s}\left(z^{\prime }\right)$ denoted the synthesis transform applied to $z^{\prime }$, while $C_{m}\left(y^{\prime }\right)$ represented the incorporation of contextual data into the probability model.

Due to the absence of prior information about $z^{\prime }$, the entropy decomposition prior model θ was used to parameterize the distribution of $z^{\prime }$ as $p_{y^{\prime }|\theta }$:

(13) $$p_{y^{\prime }|\theta }=\theta \left(z^{\prime }\right)$$

Finally, asymmetric numeral systems (ANS) [41] was employed to perform entropy coding on $y^{\prime }$ and $z^{\prime }$ compressing them into a bitstream in a lossless manner. This process produced the compressed image frame ${\hat{P}}_{i}^{\prime }$.

(14) $$P_{i}^{\prime }=\left(\left(s_{0}\times Scale\right)+C_{y^{\prime }}\right)\times Scale+C_{z^{\prime }}$$

where $s_{0}$ was the initial state of the encoder, and $C_{y^{\prime }}$ and $C_{z^{\prime }}$ were the cumulative probability over $y^{\prime }$ and $z^{\prime }$, respectively. Scale was the maximum possible value of the state and was used to control the range and precision of the state in the encoding process.

### 3.4. Video Compression Module

This paper employed a bidirectional compression module to compress the intermediate frames in each video frame sequence. The module consisted of four main components: motion information computation, motion compensation, residual calculation, and frame reconstruction as illustrated in Figure 3C. During the video compression process, bidirectional motion estimation techniques were utilized to accurately calculate the dynamic changes between the current frame and its neighboring frames.

#### 3.4.1. Motion Information Computation

The module comprised two components: the motion estimation module and the M/R compression module. The motion estimation module was responsible for calculating the bidirectional optical flow, while the M/R compression module focused on compressing this optical flow data.

**Figure 5:**
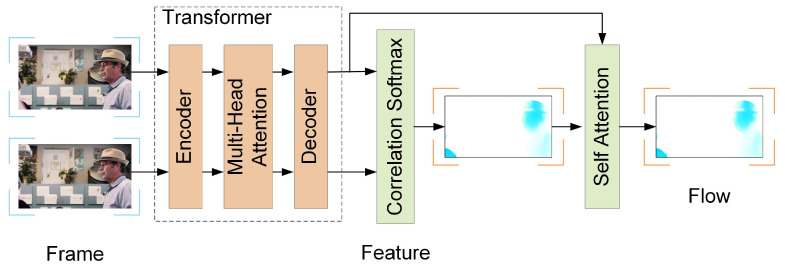
Motion estimation module. This module first extracted features from the input images and then processed these features using a transformer layer that integrated a multi-head attention mechanism to enhance the discriminative characteristics of the data. A correlation Softmax layer was employed to compute the optical flow. Finally, the self-attention layer further refined the optical flow.

Firstly, the structure of the motion estimation primarily consisted of three components: a transformer layer [42] for feature extraction and enhancement, a correlation Softmax layer for global feature matching, and a self-attention layer for refining the optical flow. The architecture of this network was illustrated in Figure 5.

Since the input bidirectional images underwent the same processing, we denoted the two adjacent frames as $P_{1}$ and $P_{2}$. After inputting $P_{1}$ and $P_{2}$, we first extracted features from them and downsample to obtain $FU_{1}$ and $FU_{2}$. These features were then sent to the transformer layer for feature enhancement, resulting in the enhanced features $FU_{1}^{\prime }$ and $FU_{2}^{\prime }$.

In the transformer layer, the formula for the multi-head attention mechanism was as follows:

(15) $$FU^{\prime }=MultiHead\left(Q,K,V\right)=Concat\left(head_{1},\ldots ,head_{n}\right)W^{O}$$

(16) $$head_{i}=Attention\left(Q,K,V\right)=softmax\left(\frac{QK^{T}}{\sqrt{d_{k}}}\right)V$$

where *Q*, *K*, and *V* were the query matrix, key matrix, and value matrix, respectively, which were obtained by transforming the down-sampled features FU through the encoder. $W^{0}$ denoted the linear transformation matrix.

Next, we calculated the global correlation [43] between the two frame features to obtain the global feature similarity *R* of each pixel in $FU_{1}^{\prime }$ relative to each pixel in $FU_{2}^{\prime }$:

(17) $$R=\frac{\left(FU_{1}^{\prime }\right){\left(FU_{2}^{\prime }\right)}^{T}}{\sqrt{D}}$$

where $R\in R^{H\times W\times H\times W}$ represented the feature similarity, and $\sqrt{D}$ was a normalization factor used to prevent large values from arising in the dot product operation [42].

Subsequently, the predicted optical flow was obtained through the correlation Softmax layer. This layer first normalized the feature correlation *R* using a Softmax [44] to derive the transformation relationship N∈(0,1) between the pixels of the two features:

(18) $$N=softmax\left(R\right)=\frac{e^{R_{i}}}{\sum_{i=1}^{n}e^{R_{i}}}$$

where $N\in R^{H\times W\times H\times W}$ represented the transformation relationship between the features, and $R_{i}$ denoted the correlation between each point in the features, with *i* indicating any point in the feature map.

Then, by performing a weighted average of the matching probabilities of the two-dimensional pixel coordinates, we found the correspondence $N^{\prime }$ between the feature maps:

(19) $$N^{\prime }=NP$$

where $P\in R^{H\times W\times 2}$ represented the position coordinates of all pixels in the feature map.

Finally, the optical flow result was obtained by calculating the difference in corresponding pixel coordinates.

(20) $$Flow=N^{\prime }-P$$

Additionally, occlusion could prevent the identification of similar pixels during correlation matching. To address this issue, a self-attention layer was introduced, which calculated the self-similarity between images or features. This further optimized the final optical flow estimation result $Flow_{final}$:

(21) $$Flow_{final}=softmax\left(\frac{\left(FU_{1}^{\prime }\right){\left(FU_{2}^{\prime }\right)}^{T}}{\sqrt{D}}\right)Flow$$

**Figure 6:**
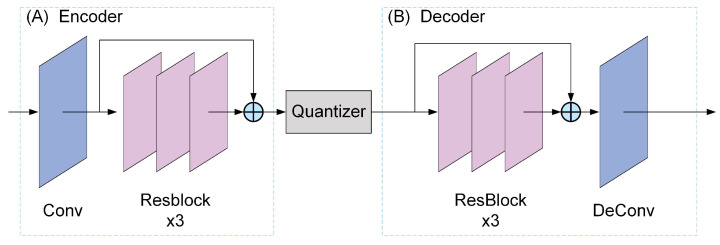
M/R compression modules. (**A**) Encoder module that consisted of a convolutional layer and a series of residual blocks; (**B**) Decoder module that reconstructed the quantized latent space data to obtain the compressed motion or residual. A quantizer converted the continuous data in the latent space into a discrete representation.

Then, the output results of motion estimation were compressed by the M/R compression module. The structure of this module was shown in Figure 6. The encoder and decoder were both composed of a set of Resblocks [45]. The motion vector or residual was transformed into a latent space by the encoder, which consisted of three Resblocks. A quantizer was employed to convert the continuous data in the latent space into discrete representations. Then, the decoder converted the quantized latent representation to reconstruct the compressed motion vector or residual.

In the Resblock, the output $x_{K}$ of the *K*-th layer could be expressed as:

(22) $$x_{K}=x_{k}+\sum_{i=k}^{K-1}F\left(x_{i},W_{i}\right)$$

where $x_{k}$ represented the output of any layer *k* that was shallower than *K*, and $F(x_{i},W_{i})$ performed a nonlinear transformation on $x_{i}$, where $W_{i}$ was the weight of that layer.

The final motion vector $M_{v}$ for the adjacent frames was represented as:

(23) $$M_{v}=Dec\left(Q\left(Conv\left(Flow_{final}\right)+\sum_{i=0}^{2}F\left(x_{i},W_{i}\right)\right)+\sum_{i=3}^{5}F\left(x_{i},W_{i}\right)\right)$$

where $Conv(Flow_{final})$ represented one convolution operation on $Flow_{final}$, Dec denoted the inverted convolutional layer, *Q* represented the quantizer.

#### 3.4.2. Motion Compensation

**Figure 7:**
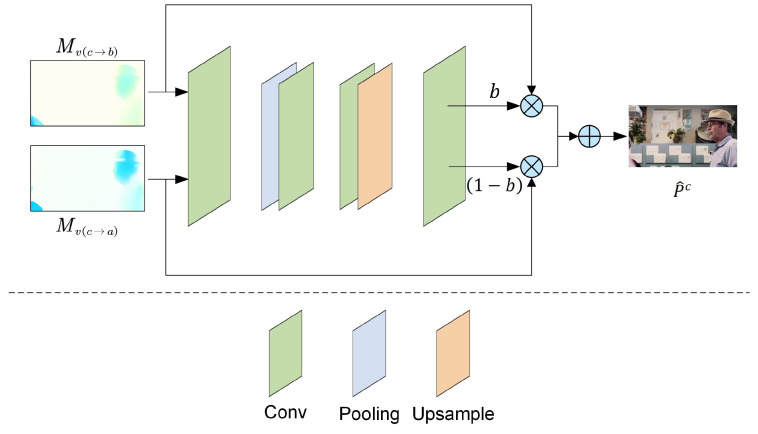
Motion compensation module. The inputs were the forward motion information $M_{v(c\to b)}$ and backward motion information $M_{v(c\to a)}$, while the output ${\hat{P}}_{c}$ was the reconstructed masked image.

In this context, we defined the three frames involved in motion compensation, processed in temporal order, as before (b), current (c), and after (a). The structure of bidirectional motion compensation was illustrated in Figure 7. Using bidirectional optical flow estimates $M_{v(c\to b)}$ and $M_{v(c\to a)}$, the before frame $P_{b}$ and the after frame $P_{a}$ were transformed onto the current frame $P_{c}$ through bilinear mapping. This process integrated information from both the before and after frames into the current frame. Finally, the predicted frame ${\hat{P}}_{c}$ was computed using the obtained masks along with the reference frames from before and after:

(24) $${\hat{P}}_{c}=b\cdot M_{v(c\to b)}+\left(1-b\right)\cdot M_{v(c\to a)}$$

where *b* denoted the pixel-level masking coefficient for frame ${\hat{P}}_{c}$, $M_{v(c\to b)}$ represented the motion vector from the current frame to the before frame, and $M_{v(c\to a)}$ indicated the motion vector from the current frame to the after frame.

#### 3.4.3. Residual Calculation

Considering that the final predicted frame ${\hat{P}}_{c}$ still had some deviation from the original frame $P_{c}$, we calculated the difference between these two frames. Subsequently, the M/R compression module was utilized to compress this difference, resulting in the final residual Ress.

(25) $$Res_{\left(x,y\right)}=C_{m/r}\left({\hat{P}}_{c(x,y)}-P_{c(x,y)}\right)$$

where $Res_{\left(x,y\right)}$ denoted the residual value at position (x,y), while $P_{c(x,y)}$ denoted the pixel value of the original frame at position (x,y). ${\hat{P}}_{c(x,y)}$ denoted the pixel value of the predicted frame at position (x,y).

#### 3.4.4. Frame Reconstruction

The final reconstructed frame $P_{c}^{\prime }$ was composed of the bidirectional motion information $M_{v(c\to b)}$, $M_{v(c\to a)}$, and residuals of that frame Res:

(26) $$P_{c}^{\prime }=M_{v(c\to b)}+M_{v(c\to a)}+Res$$

### 3.5. Loss Function

To minimize the required bit rate during video encoding while maximizing the quality of decoded frames, the loss function is defined as follows:

(27) $$L=\alpha \left[R_{i}+\lambda_{i}d\left(P_{i}-{\hat{P}}_{i}^{\prime }\right)\right]+\beta \left[R_{m}+R_{r}+\lambda_{b}d\left(P_{c}-P_{c}^{\prime }\right)\right]$$

where $R_{i}$, $R_{m}$, and $R_{r}$ represented the bit counts for encoding the first and last frames, the motion vectors, and the residuals, respectively. The term $d(P_{i}-{\hat{P}}_{i}^{\prime })$ indicated the distortion between the input frame $P_{i}$ and the reconstructed frame ${\hat{P}}_{i}^{\prime }$ in the image compression module, while $d(P_{c}-P_{c}^{\prime })$ represented the distortion between the input frame $P^{c}$ and the reconstructed frame $P_{c}^{\prime }$, where d(·) was the MSE. The parameters $\lambda_{i}$ and $\lambda_{b}$ were hyperparameters to control the rate-distortion trade-off. The coefficients α and β managed the training phase: when α=1 and β=0, only the image compression module was trained; when α=0 and β=1, only the video compression module was trained.

### 3.6. Evaluation Metrics

To evaluate the compression performance of the model, we assessed the peak signal-to-noise ratio (PSNR) [46] and the multi-scale structural similarity index (MS-SSIM) at different bit rates (bits per pixel, bpp). The primary evaluation method involved comparing the distortion between the reconstructed frames and the original frames. Additionally, we analyzed the compression efficiency of different models using the Bjøntegaard-Delta Rate (BD-rate).

PSNR served as a metric for measuring the quality of signal reconstruction in fields such as image compression, representing the ratio of peak signal energy to average noise energy. The calculation was as follows:

(28) $$PSNR=10log_{10}\left(\frac{{\left(2^{n}-1\right)}^{2}}{MSE}\right)$$

(29) $$MSE=\frac{1}{H\times W}\sum_{i=1}^{H}\sum_{j=1}^{W}{\left[X\left(i,j\right)-Y\left(i,j\right)\right]}^{2}$$

where *n* represented the number of bpp, typically set to 8, which resulted in 256 grayscale levels for each pixel. X(i,j) and Y(i,j) denoted the pixel values at corresponding coordinates, while *H* and *W* indicated the height and width of the image, respectively. Mean squared error (MSE) [47] measured the discrepancy between the predicted values and the actual values. In this paper, we calculated the average PSNR of all frames in each video sequence as the final result.

MS-SSIM functioned as an image quality assessment metric that evaluated image similarity based on three aspects: luminance, contrast, and structure. The results of the MS-SSIM assessments aligned more closely with subjective quality evaluations. The formula was as follows:

(30) $$MS\_SSIM\left(x,y\right)={\left[l_{M}\left(x,y\right)\right]}^{\alpha_{M}}\cdot \prod_{j=1}^{M}{\left[c_{j}\left(x,y\right)\right]}^{\beta_{j}}\cdot {\left[s_{j}\left(x,y\right)\right]}^{\gamma_{j}}$$

where *x* and *y* represented the two images being compared, while $l_{M}\left(x,y\right)$, $c_{j}\left(x,y\right)$, and $s_{j}\left(x,y\right)$ denoted the distributions of similarity for luminance, contrast, and structure, respectively. The exponents $\alpha_{M}$, $\beta_{j}$, and $\gamma_{j}$ served as weight parameters to adjust the relative importance of these three components. *M* represented the number of iterations. In this paper, we calculated the average MS-SSIM of all frames in each video sequence as the final result.

BD-rate was a method used to evaluate the performance of video encoders, quantifying the bitrate difference between two encoders within a specific quality range. The formula was as follows:

(31) $$BDrate=\frac{\int_{Q_{min}}^{Q_{max}}\left(f_{2}\left(Q\right)-f_{1}\left(Q\right)\right)dQ}{\int_{Q_{min}}^{Q_{max}}f_{1}\left(Q\right)dQ}\times 100\%$$

where *Q* was the quality metric, denoted the PSNR value of the image after compression by the encoder. f(·) represented the interpolation function, expressed as $f\left(Q\right)=10^{a\cdot Q+b}$, where *a* represented the sensitivity of quality *Q* to the image bitrate, while *b* was a constant term that reflected the baseline level of the model and was related to the baseline value of the bitrate. $f_{1}\left(Q\right)$ and $f_{2}\left(Q\right)$ were the interpolation functions for the two encoders being compared.

Furthermore, the study compared its model against several baseline methods:

- DVC: This model is the first end-to-end deep learning video compression framework, providing a promising structure for the application of deep neural networks in video compression. It is commonly regarded as a benchmark algorithm in the field of deep learning video compression [15].
- OpenDVC: This model reproduced and optimized DVC using TensorFlow. It not only replicated DVC’s PSNR optimization model but also offered an MS-SSIM-optimized DVC model [48].
- RLVC: This method featured a macro encoding framework similar to existing deep video compression techniques. It was one of the earlier approaches to compress the current frame by utilizing temporal correlations between the previous and current frames, akin to the use of adjacent frame correlations in our work [16].
- FVC: This framework performed all major operations in the feature space of images. It was chosen for comparison to verify the advantages and characteristics of our video compression algorithm in feature space processing [34].
- LHBDC: Similar to our work, this model employed a layered bidirectional video codec, combining the benefits of layered motion compensation prediction and end-to-end optimization [49].
- H.264/AVC and H.265/HEVC: H.264 and H.265 were the two widely used video compression standards at the time, serving as benchmark algorithms in the field. Comparing our algorithm with them allowed for a direct assessment of performance levels, particularly in terms of compression efficiency and video quality [32,33].

## 4. Results

### 4.1. Experimental Settings

All experiments were conducted on the same platform using Python and PyTorch [50]. The configuration of the experimental platform included a V100-SXM2 32 GB GPU, 32 GB of memory, an Intel(R) Xeon(R) Platinum 8255C CPU @ 2.50 GHz with 12 cores, and 43 GB of RAM, running Ubuntu 16.04.

The training process was divided into two stages. In the first stage, the image compression module was trained independently for 2 million steps with a learning rate of 5 × 10^−5^. In the second stage, a bidirectional compression module was trained for an additional 3 million steps using the same learning rate. Subsequently, the learning rate was reduced to R 5 × 10^−6^ for 100,000 steps of optimization. During the training process, the batch size was set to 1, and the λ values were set to (0.0035, 0.0067, 0.0130), with the Adam optimizer [51] being employed.

### 4.2. Compression Performance Comparison

In this study, we compared the PSNR, MS-SSIM and BD-rate of the different models, which offered a comprehensive assessment of the models’ performance across different levels of compression.

**Figure 8:**
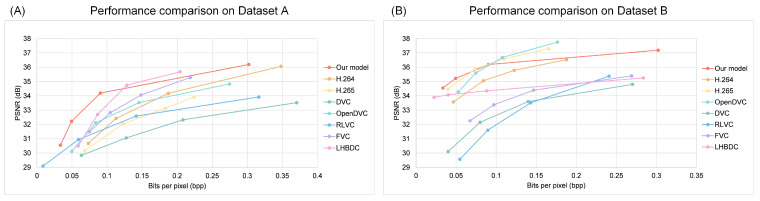
A comparison of the PSNR performance at different bpp values. (**A**) Comparison of the PSNR performance on the Dataset A. (**B**) Comparison of the PSNR performance on the Dataset B.

Initially, we evaluated the models’ PSNR results for different bpp values (0<bpp<0.4) on Dataset A and Dataset B, respectively, and compared these results with those from other video compression methods, as illustrated in Figure 8. On Dataset A, it was observed that our model outperformed most other video compression methods under the same bpp conditions, as shown in Figure 8A. This indicated that while maintaining a similar compression rate, our model generated video quality that was closer to the original. Specifically, when bpp<0.12, the PSNR values of our model ranged between 30.5 and 34.5. In contrast, when bpp>0.21, the PSNR values exceed 35, which is higher than all other methods. This meant that at higher compression rates, our approach provided the best video quality. However, within the range of 0.12<bpp<0.21, our method fell short by only 1.7% compared to the LHBDC model. On dataset B, the rate-distortion curves of our model were compared with other video compression methods, as shown in Figure 8B. The results indicated that our model outperformed most techniques. When bpp<0.07, our model surpassed all other models. However, when bpp>0.1, its performance declined by 18.17% compared to H.265 and by approximately 20% compared to OpenDVC. This suggested that our model had certain limitations when handling high-resolution videos. Nevertheless, it still demonstrated a competitive edge over other models.

**Figure 9:**
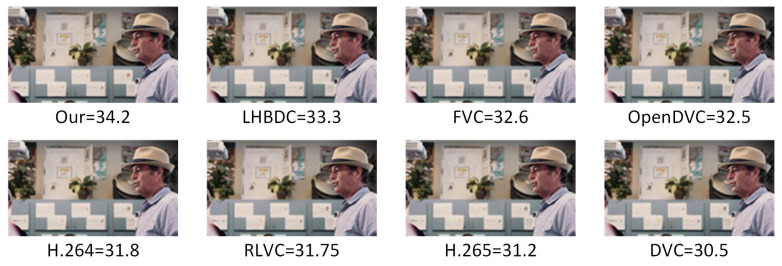
Visual effects and PSNR results.

Additionally, we presented a visual comparison of the compression results of our model and other models on the same sequence of frame images at bpp=0.1, as shown in Figure 9. It was observed that our model achieved a higher PSNR than the other models at the same bpp, and the visual quality of the video frames exceeded that of all other models. For instance, compared to DVC, our model improved PSNR by 3.7 dB. Additionally, compared to LHBDC, our method demonstrated a PSNR improvement of 0.9 dB.

**Figure 10:**
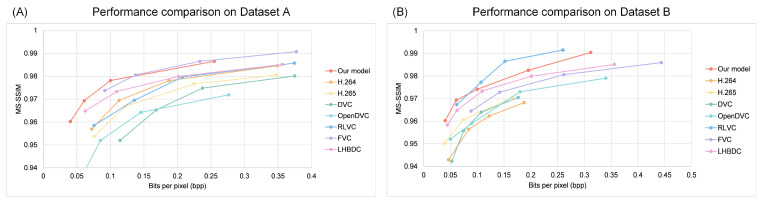
A comparison of the MS-SSIM performance at different bpp values. (**A**) Comparison of the MS-SSIM performance on the Dataset A. (**B**) Comparison of the MS-SSIM performance on the Dataset B.

Then, we evaluated the models’ performance in terms of MS-SSIM on Dataset A and Dataset B, respectively, as shown in Figure 10. The results indicated that our model achieved a higher compression rate on both Dataset A and B compared to other video compression methods. On Dataset A, when bpp>0.13, our method reduced compression by approximately 8.4% compared to FVC. At this point, the performance of our model was comparable to that of other video compression models, such as DVC, HLVC, and RLVC. At bpp=0.1, our model improved by 0.024 over the earliest DVC model and by 0.01 compared to RLVC, which also utilized a pre-trained optical flow estimation network. On dataset B, our model outperformed most other models. Specifically, at a higher compression rate bpp=0.05, our model achieved an MS-SSIM score that was 0.02 higher than that of the earliest proposed DVC. When bpp>0.83, our model was only 1.02% lower than RLVC but still outperformed other models. At bpp=0.3, our model achieved the best image quality, with an MS-SSIM score of 0.989. These results demonstrated that our method had significant advantages in compression performance.

**Figure 11:**
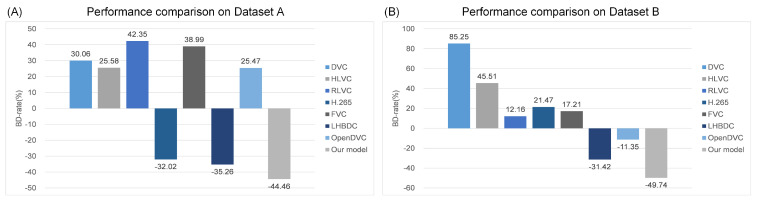
BD-rate reduction of different model. (**A**) Comparison of the BD-rate performance on the Dataset A. (**B**) Comparison of the BD-rate performance on the Dataset B.

In addition, we conducted a quantitative analysis of the BD-rate for different models at the same quality level, as shown in Figure 11. Using the compression performance of H.264 as a benchmark, the results demonstrated that our proposed method achieved a BD-rate reduction of 44.46% on Dataset A and 49.74% on Dataset B. This confirmed that our method provided a more substantial BD-rate reduction compared to other approaches, while maintaining the same compression quality.

### 4.3. Ablation Study

To validate the effectiveness of this network, we conducted an ablation experiment in which we removed the global feature-matching module and instead matched pixels from adjacent frames to derive the motion estimation results. The impact of global feature matching compared to pixel matching on PSNR and MS-SSIM was summarized in Table 1.

The results indicated significant differences in the performance of compressed output images at varying bpp levels. For Dataset A, the specific results are as follows: at bpp=0.05, PSNR dropped by 10.2% and MS-SSIM by 18.7%; at bpp=0.1, PSNR decreased by 13.8% and MS-SSIM by 19.1%; at bpp=0.15, PSNR was reduced by 10.4% and MS-SSIM by 15.9%; at bpp=0.2, PSNR decreased by 10.8% and MS-SSIM by 14.5%; at bpp=0.25, PSNR was reduced by 13.8% and MS-SSIM by 19.1%; and at bpp=0.3, PSNR was reduced by 8.5% and MS-SSIM by 9.4%.

The comparison for Dataset B revealed even more pronounced results: at bpp=0.05, PSNR was reduced by 22.9% and MS-SSIM by 26.1%; at bpp=0.1, PSNR decreased by 19% and MS-SSIM by 24.7%; at bpp=0.15, PSNR was reduced by 17.3% and MS-SSIM by 18.1%; at bpp=0.2, PSNR dropped by 14.9% and MS-SSIM by 15.6%; at bpp=0.25, PSNR decreased by 13.8% and MS-SSIM by 15.2%; and at bpp=0.3, PSNR was reduced by 13.8% and MS-SSIM by 15.2%.

These results demonstrated that global feature matching outperformed pixel-matching motion estimation in terms of image compression performance. This phenomenon could be attributed to the global feature-matching network’s ability to capture richer contextual information, allowing it to more effectively represent and reconstruct the overall structure of the image.

## 5. Discussion

In this study, we proposed DeepBiVC, an innovative bidirectional coding video compression scheme aimed at enhancing video compression efficiency. DeepBiVC adopted a two-stage learning strategy: the first stage focused on compressing the first and last frames using an image compression module, while the second stage addressed the compression of intermediate frames through a video compression module. The image compression module employed an INN to achieve efficient compression while minimizing information loss and maintaining image quality. Meanwhile, the video compression module utilized global feature matching-based optical flow estimation, effectively capturing inter-frame motion information by explicitly transforming the optical flow problem into a matching process between deep features.

Compared to existing video compression standards and computational algorithms, significantly improving compression efficiency while maintaining high-quality video output. This performance enhancement can be attributed not only to the efficient compression capabilities of INN but also to the optical flow estimation method based on global feature matching, which effectively captured inter-frame motion information and reduced redundant data. These combined technologies make DeepBiVC highly promising for practical applications.

However, the model still had some shortcomings:

First, the inference speed of the model was relatively slow, primarily due to the incorporation of the INN and global optical flow estimation modules. The INN model involved complex reversible computation operations, while global optical flow estimation required precise analysis of pixel motion between adjacent frames. These two factors consumed significant computational resources, impacting the inference speed.

Second, the training dataset was limited in both scale and scope compared to real-world video data. This limitation may have resulted in suboptimal performance when handling more diverse and broader video datasets.

Lastly, the current model was relatively large, posing challenges for deployment on embedded devices such as network cameras and dashcams, thus limiting its practical applications.

## 6. Conclusions

In this paper, we presented a novel bidirectional video compression model, DeepBiVC, based on an image compression module and a video compression module. The image compression module utilized an INN to better represent image compression as a reversible process, thereby reducing information loss during compression and lowering the model’s parameter count. Additionally, the video compression module employed global matching-based optical flow estimation, reformulating optical flow as an explicit matching problem between two sets of deep features.

The experimental results indicated that DeepBiVC achieved a PSNR of 36.05 and an MS-SSIM of 0.98 on Dataset A with bpp=0.3. On Dataset B and bpp=0.3, DeepBiVC obtained a PSNR of 37.16 and an MS-SSIM of 0.98. The performance of DeepBiVC surpassed that of state-of-the-art image compression methods under the same conditions. Future research may explore model compression and quantization techniques to minimize storage requirements and reduce computational overhead. Efforts will also prioritize optimizing inference speed and tailoring the model for specific hardware, such as embedded devices, to enhance deployment and ensure effective performance across diverse application scenarios.

## Figures and Tables

**Table 1:** The impact of global feature matching and pixel matching on PSNR and MS-SSIM.

		Global Feature Matching		Pixel Matching	
Dataset A	bpp	PSNR	MS-SSIM	PSNR	MS-SSIM
	0.05	31.57	0.964	28.36	0.784
	0.10	33.92	0.978	29.25	0.791
	0.15	34.43	0.981	30.84	0.825
	0.20	34.97	0.984	31.17	0.841
	0.25	35.51	0.986	32.59	0.884
	0.30	36.05	0.989	32.98	0.896
Dataset B	0.05	35.21	0.964	27.15	0.712
	0.10	36.23	0.974	29.33	0.733
	0.15	36.46	0.979	30.17	0.801
	0.20	36.69	0.982	31.22	0.829
	0.25	36.93	0.986	31.82	0.836
	0.30	37.16	0.989	32.03	0.877

## Data Availability

Data is contained within the article.
